# A Novel Method for Differential Prognosis of Brain Degenerative Diseases Using Radiomics-Based Textural Analysis and Ensemble Learning Classifiers

**DOI:** 10.1155/2021/7965677

**Published:** 2021-08-05

**Authors:** Manju Jain, C. S. Rai, Jai Jain

**Affiliations:** ^1^University College of Information, Communication and Technology, Guru Gobind Singh Indraprastha University, Dwarka Sector 16-C, New Delhi 110078, India; ^2^Meerabai Institute of Technology Maharani Bagh, New Delhi 110065, India; ^3^Media Agility India Ltd, New Delhi, India

## Abstract

We propose a novel approach to develop a computer-aided decision support system for radiologists to help them classify brain degeneration process as physiological or pathological, aiding in early prognosis of brain degenerative diseases. Our approach applies computational and mathematical formulations to extract quantitative information from biomedical images. Our study explores the longitudinal OASIS-3 dataset, which consists of 4096 brain MRI scans collected over a period of 15 years. We perform feature extraction using Pyradiomics python package that quantizes brain MRI images using different texture analysis methods. Studies indicate that Radiomics has rarely been used for analysis of brain cognition; hence, our study is also a novel effort to determine the efficiency of Radiomics features extracted from structural MRI scans for classification of brain degenerative diseases and to create awareness about Radiomics. For classification tasks, we explore various ensemble learning classification algorithms such as random forests, bagging-based ensemble classifiers, and gradient-boosted ensemble classifiers such as XGBoost and AdaBoost. Such ensemble learning classifiers have not been used for biomedical image classification. We also propose a novel texture analysis matrix, Decreasing Gray-Level Matrix or DGLM. The features extracted from this filter helped to further improve the accuracy of our decision support system. The proposed system based on XGBoost ensemble learning classifiers achieves an accuracy of 97.38%, with sensitivity 99.82% and specificity 97.01%.

## 1. Introduction

Medical image processing has travelled a long journey since the last two decades. The past decade has seen the bridging of medical and information technology. It led to the development of decision support systems for early identification of various brain diseases. Age and structural changes in brain cause physiological alterations, which are reflected in routine human behaviour [1, 2]. Along the years, various studies and constant attempts have been made to study dementia.

Studies [3–5] focus on specific regions of interest in brain volumes, and these are calculated from two dimensional manually traced areas. Segmentation algorithms are used to segment out gray matter (GM), white matter (WM), and cerebrospinal fluid (CSF). Such volumetric studies are limited to known brain structures like hippocampus and amygdala, perirhinal, entorhinal, and parahippocampal cortex.

Voxel-based studies [6–8] provide an alternative neuroimaging method. These studies apply a general linear model (GLM) to each voxel of an MRI and statistically compare them with standard voxel values using Jacobean matrices.

Many studies [9, 10] give detailed insights on comparisons between voxel based and volumetric studies.

Several studies [11–13] use cortical thickness measurement as a biomarker for the process of identification of various brain aging diseases.

With the advancements in the machine learning techniques for image processing and image analysis and the availability of abundance of medical imaging data, medical informatics [14, 15] has achieved great heights. The workshop MICCAI 2014 “Challenges of Computer aided diagnosis of Dementia on Structural MRI data” addresses the challenges of applying different algorithms on the same data and the same algorithm on different data. A summary of all algorithms presented in MICCAI 2014 is listed in [16]. This paper did a standardized comparison of different studies in the domain of the computer-aided decision support system for the identification of dementia-related diseases using structured MRI data. The best performing algorithm yielded an accuracy of 63% and receiver operating characteristics area under the curve with value 78.8%.

A review of various studies used for brain disorder detection using the machine learning techniques is published in [17]. Another review of the latest image processing techniques for studying brain pathology is summarized in [18].

A set of studies have been done on how oxygen supply changes the brain functioning [19, 20].

The functional modalities of medical imaging include MRI (magnetic resonance imaging), PET (positron emission tomography), and CT (computerized tomography) giving us an insight about the pathophysiology of the organ under observation. Radiologists analyze this information with their experience and knowledge. They find this time consuming and cumbersome. In this study, we explore machine learning techniques to analyze data extracted from medical images. Machine learning is the study of algorithms that solve a problem by leaning from underlying patterns in data, as opposed to statistical heuristics or rule-based programming. Radiomics [21, 22] aids in extracting imaging-based statistical biomarkers from medical images which can be used as features for machine learning methods to get accurate predictions. Ageing leads to degeneration of the brain, which may lead to dementia, further precipitating such diseases like Alzheimer's dementia, vascular dementia, dementia with lewy body dementia, posterior cortical atrophy, and front temporal lobar degeneration. These diseases affect different regions of the brain. Clinical Dementia Rating or CDR is a five-point scale to stage dementia, ranging from 0 to 3, where 0 denotes no pathological degeneration (control patients) while any value greater than 0 indicates some pathological brain degeneration (test patients).

In this study, we propose a novel approach to develop a computational decision support system capable of differentiating control patients from test patients by analyzing features of their MRI images using Radiomics. This system can be used to assist radiologists for fast and accurate decisions.

1. We explore the OASIS-3 dataset [23], which is a longitudinal dataset with 4096 MRI scans. This dataset also gives specific details about how the CDR value changes for a subject with respect to changes in the subject's MRI scan. These ratings can be used to label the MRI scans as healthy scans or scans showing signs of brain degeneration. Using these labels for a scan, a supervised machine learning binary classifier can be trained to support brain degeneration prognosis
2. We employ data preprocessing best practices such as data augmentation and feature selection which help to mitigate overfitting and underfitting of the classifier and drive it to achieve optimal accuracy on our data
3. Feature extraction is done using Pyradiomics, which provides a python implementation of the study [24]. Pyradiomics provides a unified and standardized set of features from structured MRIs based on shape and volume as well as texture-based statistical features. Advanced Pyradiomics algorithms can handle missing data in case of low resolution MRI scans. Literature studies indicate that Radiomics has mostly been explored for oncological studies [25, 26], but not for understanding brain cognition. Our study is also an effort to determine the efficiency of Radiomics features from structural MRI scans for classification of brain degeneration diseases
4. We explored various ensemble learning classification algorithms such as random forests, bagging-based ensemble classifiers, and gradient-boosted ensemble classifiers such as XGBoost and AdaBoost for our classification tasks. Such ensemble learning classifiers have not been used for biomedical image classification
5. We propose a novel image texture analysis filter, Decreasing Gray-Level Matrix, which further improves the performance of our ensemble learning classifiers

We conclude the paper by comparing our novel solution with existing work in this field. Our results show that the proposed solution outperforms existing studies on various performance metrics such as accuracy, specificity, and sensitivity.

## 2. Materials and Methods

### 2.1. Data Acquisition

**Figure 1:**
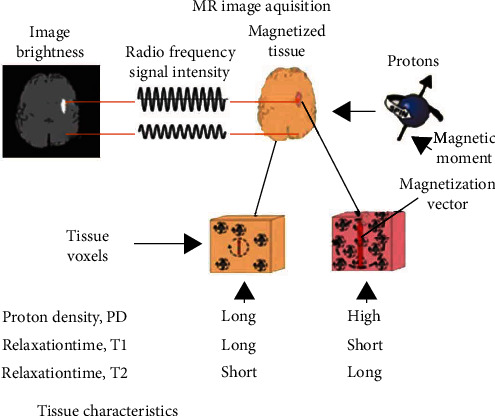
MRI acquisition process.

Magnetic resonance imaging is the process of acquiring images of anatomical structures using magnetic field and radio frequency signals to detect diseases and functional problems. The “image is snapped” with different contrasts as different tissues and fluids react differently to magnetization signals. Tissue demagnetization time is different for different tissues. These times are identified as T1 and T2. Another characteristic of a tissue that affects an MRI is its proton density known as PD.  Figure 1 depicts the complete MRI acquisition process.

In our study, we used the latest OASIS-3 dataset [23], which is an open source brain MRI database published in 2019. Most of the earlier studies have been done using ADNI datasets, which are cross-sectional datasets and do not include more than 500 subjects. OASIS-3 is the largest longitudinal dataset of longitudinal MRI images that consists of 1068 subjects (age group of 46 to 95), collected over a period of 15 years.

“The CDR is a 5-point scale used to characterize six domains of cognitive and functional performance applicable to Alzheimer disease and related dementias: Memory, Orientation, Judgment & Problem Solving, Community Affairs, Home & Hobbies, and Personal Care. The necessary information to make each rating is obtained through a semi-structured interview of the patient and a reliable informant or collateral source (e.g. family member)” [27].

The OASIS database also provides CDR for each subject. The CDR values of a person over a particular period of time may or may not be the same. There are multiple scans of the same subject (4-5 times) in the time period of 15 years with different CDR values. These scans can be further used as samples. Hence, the database has more than 4000 MRIs.

### 2.2. Data Preprocessing

**Figure 2:**
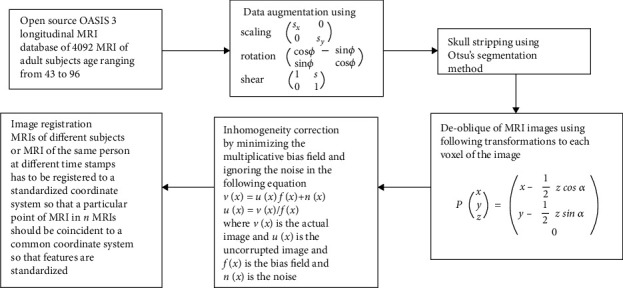
Data preprocessing flow.

We performed data preprocessing using Python and FreeSurfer [28]. Main steps of data preprocessing are listed below and more visually shown in Figure 2.

#### 2.2.1. Data Augmentation

We augmented our data to make our classifier much more tolerant towards variance in the data (prevents overfitting) and to increase dataset size (prevents underfitting). We employed 4 augmentation techniques:

1. *Flips*. Each image is flipped horizontally as well as vertically.
2. *Scaling*. Each image is scaled in either “*x*” or “*y*” direction with the help of a transform matrix $\left(\begin{array}{c} s_{x}\;0\\ 0\;s_{y} \end{array}\right)$.
3. *Rotations*. Affine transform matrix $\left(\begin{array}{c} \cos\emptyset -\sin\emptyset \\ \sin\emptyset \cos\emptyset \end{array}\right)$ gives rotated MRI images in different directions. “∅” was varied between 25 and 195.
4. *Shears*. Affine transform matrix $\left(\begin{array}{c} 1\;s\\ 0\;1 \end{array}\right)$ applied to each image where Shear value changes from 0.3 to 0.7.

#### 2.2.2. De-Oblique

During the MRI process, the subject's head may be tilted from to cover the whole brain or to avoid artefacts caused by water and air in the nose and eyes of the subject. This causes the MRI to be oblique and makes intersubject or intrasubject registration more difficult. The MRI images in our dataset were de-obliqued using the FreeSurfer software.

#### 2.2.3. Inhomogeneity Correction

Brain consists of different types of tissues like gray matter (GM), white matter (WM), and cerebrospinal fluid (CSF), and all these tissues have different range of penetration to the magnetic field and may result into very bright or very dull artefacts in the MRI images. This may confuse a radiologist since all tissues of a particular type should have exact intensity and brightness values. The process of correcting this is known as inhomogeneity correction.

#### 2.2.4. Skull Stripping

The nonbrain parts (skull, neck, eyes, and nose) were removed from all MRI images to have a uniform area of study.

#### 2.2.5. Registration

The brain consists of very fine spatial structures, due to which it is very difficult to extract and integrate the information from different images. The thickness of the cortex can be as small as 5 mm. The thickness of thalamic nuclei only extends to few millimetres. Registration is the process of aligning different MRI images in such a way that the voxels of a particular tissue from all of those images correspond to the same 3D location. We applied and adjusted the registration parameters, i.e., translations, rotations, scaling, and shear operations at voxel level to make the MRI images concurrent.

### 2.3. Feature Extraction

#### 2.3.1. Features Based on Shape

**Figure 3:**
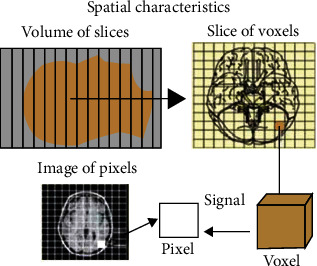
Spatial characteristics of MRI.

To extract features based on shape, we studied spatial characteristics of MRIs as depicted in Figure 3.

*Slice*: an MRI is a 3D image. It consists of a set of contiguous 2D slices. These slices may either represent the axial, sagittal, or longitudinal cross section of the subject's brain.

**Figure 4:**
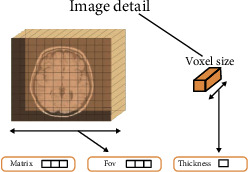
Voxel details.

*Voxel*: each slice is subdivided into rows and columns. The intersection of each row and column represents a volume of the brain. This is known as a voxel. The field-of-view matrix of a particular size of the slice is used to determine the voxel size. The voxel details are depicted in Figure 4.

**Figure 5:**
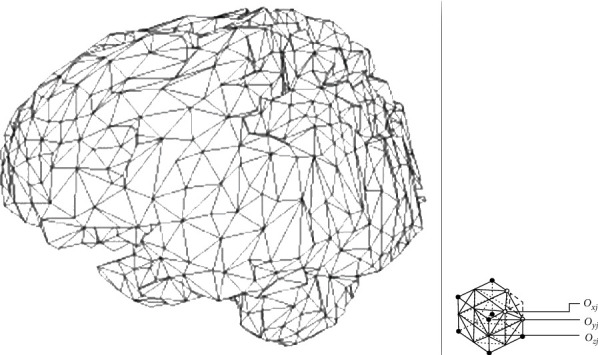
(a) Decimation of triangles as a mesh of the whole brain. (b) The trapezium points and edges. *X*_*v*_ is the number of voxels included in the masked region. *V*_*m*_ is the volume of the mesh mm^3^. *A*_*m*_ the surface area of the mesh in mm^2^.

Shape features include legends of 3D size and shape. We took the whole brain area and volume as our region of interest. A triangular mesh encapsulating the whole brain area was used to extract various shape features. Figures 5(a) and 5(b) show how a brain is treated as a mesh surface [28]. The mesh has *X*_*f*_ number of triangles.

From this meshed surface of brain, as in Figure 5(b), we calculated the following different shape features [24].

- Mesh volume *V*_*j*_ = *O*_*xi*,_.(*O*_*yj*_ × *O*_*zj*_)/6 (*O*_*xi*,_*O*_*yj*,_*O*_*zj*_ are the tetrahedral vertices)

(1) $$\begin{array}{c} V_{m}=\overset{X_{f}}{\underset{j=1}{\sum }}V_{j}. \end{array}$$

(ii) *Voxel Volume*. Voxel_volume_ = ∑_*j*=1_^*X*_*v*_^*V*_*m*_

The whole brain volume can be obtained by multiplying the voxel volume *V*_*j*_ with the number of voxels in the brain.

- *Surface Area*. *A*_*J*_ = 1/2(*X*_*j*_*Y*_*j*_ × *X*_*j*_*Z*_*j*_)

(2) $$\begin{array}{c} A_{m}=\overset{X_{f}}{\underset{j=1}{\sum }}A_{j}. \end{array}$$

To calculate the surface area of the whole brain, it is divided into small mesh areas. We first calculate the surface area of each mesh and then sum all of them.

- *Ratio of the Surface Area to the Volume of the Brain*. *A*_*m*_/*V*_*m*_
- Lower the ratio more the compactness
- *Maximum 3D Diameter*. It is the largest Euclidean distance on the various mesh surfaces on the whole brain.
- *The Maximum 2D Diameter of the Slice*. It is the defined as the largest Euclidean distance on the whole brain mesh surfaces where mesh vertices are in the axial plane.
- *Major Axis length*. $4\sqrt{\gamma_{\text{major}}}$
- This feature calculates the largest axis length of the whole brain area
- *Minor Axis Length*. $4\sqrt{\gamma_{\text{minor}}}$
- This feature represents the minimum axis length of the whole brain area
- *Elongation*. This feature gives the relationship between the largest and smallest component of the whole brain.
- $\text{Elongation}=\sqrt{\gamma_{\text{minor}}/\gamma_{\text{major}}}$
- $\text{Flatness}=\sqrt{\gamma_{\text{least}}/\gamma_{\text{major}}}$

#### 2.3.2. First-Order Features

These features are obtained by the statistical analysis of the whole brain based on values of voxel intensities [24].

Let *S* be a set of *N*_*v*_ voxels in the whole brain.

Let *N*_*v*_ be the discrete level of intensities in the whole brain then *X*(*i*) is the first-order histogram.

The normalized first-order histogram *x*(*i*) = *X*(*i*)/*N*_*v*_

- Energy = ∑_*i*=1_^*N*_*v*_^(*S*(*i*) + *c*)^2^
- Total energy=*V*_voxel_∑_*i*=1_^*N*_*v*_^(*S*(*i*) + *c*)^2^
- Entropy = −∑_*i*=1_^*N*_*d*_^*x*(*i*)log_2_(*x*(*i*)+∈)
- Minimum = min(*S*)
- 10^th^ percentile of *S*
- 90^th^ percentile of *S*
- Maximum = max(*S*)
- Mean = 1/*N*_*v*_∑_*i*=1_^*N*_*v*_^*S*(*i*) is the average gray-level intensity of the whole brain
- Median = the median gray level of the whole brain
- Range = max(*S*) − min(*S*)
- Absolute mean deviation=$\sqrt{1/N_{v}\sum_{i=1}^{N_{v}}\left|S\left(i\right)-\overset{¯}{S}\right|}$
- $\text{Root mean square value of the whole brain }\left(\text{RMS}\right)=\sqrt{1/N_{v}\sum_{i=1}^{N_{v}}{\left(S\left(i\right)+c\right)}^{2}}$
- $\text{Standard deviation}=\sqrt{1/N_{v}\sum_{i=1}^{N_{v}}{\left(S\left(i\right)-\overset{¯}{S}\right)}^{2}}$
- $\text{Skewness}=\sqrt{1/N_{v}\sum_{i=1}^{N_{v}}{\left(S\left(i\right)-\overset{¯}{S}\right)}^{3}}/{\left(\sqrt{1/N_{v}\sum_{i=1}^{N_{v}}{\left(S\left(i\right)-\overset{¯}{S}\right)}^{2}}\right)}^{3}=\mu_{3}/\sigma^{3}$
- $\text{Kurtosis}=\sqrt{1/N_{v}\sum_{i=1}^{N_{v}}{\left(S\left(i\right)-\overset{´}{S}\right)}^{4}}/{\left(\sqrt{1/N_{v}\sum_{i=1}^{N_{v}}{\left(S\left(i\right)-\overset{¯}{S}\right)}^{2}}\right)}^{2}=\mu_{4}/\sigma^{4}$
- $\text{Variance}=1/N_{v}\sum_{i=1}^{N_{v}}\left|S\left(i\right)-\overset{¯}{S}\right|$

#### 2.3.3. Gray-Level Cooccurrence Matrix [24]

**Figure 6:**
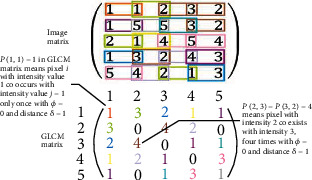
GLCM procedure. Different color schemes were used to track the steps.

GLCM is a texture filter that gives the pixel distribution of a particular set of pixels *i*, *j* in a specific direction and distance. The *p*(*i*, *j*) | ∅, *δ*| value of GLCM represents the number of times; the pixel with intensity *i* coexists with intensity *j* with angle Ø and distance *δ*.  Figure 6 shows how GLCM can be obtained from image matrix. The different color schemes indicate a particular pixel's coexistence. Generally, the following statistical features are extracted and then averaged over GLCM for each direction (angle).

- Autocorrelation
- Joint average
- Entropy
- Variance
- Contrast
- Energy
- Homogeneity
- Inverse of the difference movement
- Inverse variance

#### 2.3.4. Gray-Level Size Zone Matrix [24]

**Figure 7:**
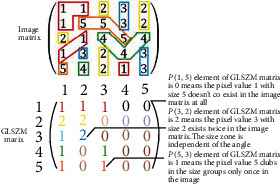
GLSZM procedure. Different color schemes were used to track the steps.

GLSZM quantifies different pixel intensity values in different size zones. A size zone is defined as connected pixels/voxels with the same gray-level irrespective of direction. The *P*(*i*, *j*) element of GLSZM represents the number of times the intensity value *i* of the size zone *j* exists in the image matrix.  Figure 7 depicts how GLSZM can be obtained from the image matrix. Different colors indicate different size zones of different intensity values.

GLSZM can be used to extract the following features:

- Emphasis on small areas
- Emphasis on large areas
- Gray-level nonuniformity
- Normalized gray-level nonuniformity
- Size zone nonuniformity
- Normalized size zone nonuniformity
- Low gray-level emphasis on small areas
- High gray-level emphasis on small areas
- Low gray-level emphasis on large areas
- High gray-level emphasis on large areas

#### 2.3.5. Gray-Level Run Length Matrix (GLRLM) [24]

**Figure 8:**
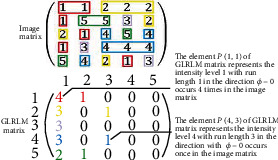
GLRLM procedure. Different color schemes were used to track.

The intensity runs in a GLRLM are defined as the length of connected pixels of equal intensity values and in a particular direction. A GLRLM element *P*(*i*, *j*) | ∅ represents the number of times a particular run length *j* of intensity *i* in direction Ø occurs in the image matrix.  Figure 8 depicts how GLRLM can be obtained from image matrix. Different colors indicate different run lengths of the particular length in a particular direction.

The GLRLM is used to extract following features:

- Emphasis on short run
- Emphasis on long run
- Nonuniform gray level
- Normalized nonuniform gray level
- Nonuniform run length
- Normalized nonuniform run length
- Run percentage
- Variance of gray level
- Run variance
- Run entropy
- Low gray-level run emphasis
- High gray-level emphasis
- Short run low gray-level emphasis
- Short run high gray-level emphasis
- Long run low gray-level emphasis
- Long run high gray-level emphasis

#### 2.3.6. Neighbouring Gray Tone Difference Matrix [24]

Here, we consider neighbouring pixels of a particular pixel at a distance *∂* of that pixel. This matrix is the set of absolute differences of the gray levels of the voxel and its neighbouring voxels. Let *P*_*nv*_ be the set of whole brain voxels; then, *p*_*nv*_(*i*_*x*_, *i*_*y*_, *i*_*z*_) belongs to *P*_*nv*_ where *p*_*nv*_ denotes the gray level of the voxel at position (*i*_*x*_, *i*_*y*_, *i*_*z*_). The average gray level of the neighbourhood is given as follows: $\overset{¯}{G_{j}}=\overset{¯}{G}\left(i_{x},i_{y},i_{z}\right)=1/v\sum_{k_{x}=-\partial }^{\partial }\sum_{k_{y}=-\partial }^{\partial }\sum_{k_{z}=-\partial }^{\partial }p_{nv}\left(i_{x}+k_{x},i_{y}+k_{y},i_{z}+k_{z}\right)$ where *V* is total number of voxels in the whole brain.

- Let *i*  denote the value of gray levels in the image
- Let *n*_*i*_ denote the number of voxels of gray level *i*
- Let *p*_*i*_ denote the gray-level probability
- Let *s*_*i*_ be the sum of absolute difference of a gray level *i*

**Figure 9:**
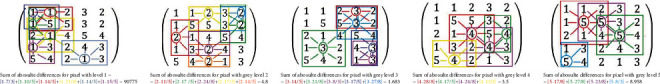
(a) NGTDM for neighbours of 1. Different color schemes were used to track NGTDM procedure to calculate the absolute sum of gray-level difference. (b) NGTDM for neighbours of 2. Different color schemes were used to track NGTDM procedure to calculate the absolute sum of gray-level difference. (c) NGTDM for neighbours of 3. Different color schemes were used to track NGTDM procedure to calculate absolute sum of gray-level difference. (d) NGTDM for neighbours of 4. Different color schemes were used to track NGTDM procedure to calculate the absolute sum of gray-level difference. (e) NGTDM for neighbours of 5. Different color schemes were used to track NGTDM procedure to calculate absolute sum of gray-level difference.

**Figure 10:**
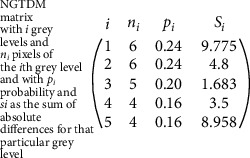
NGTDM.

Figures 9(a)–9(d) are the required NGTDM for the pixel with intensities 1-4.  Figure 9(e) describes how the absolute difference of the different gray levels is calculated. Different colors are used to track down the neighbours of a particular gray level as shown in the following example where we have 5 discrete gray levels 1 to 5.  Figure 10 is the final NGTDM.

Features calculated from NGTDM are as follows:

- The neighbourhood-based coarseness
- Neighbourhood-based contrast
- Rate of change of gray levels within voxels
- The complexity of neighbourhood gray levels
- Strength of neighbourhood gray levels

#### 2.3.7. Gray-Level Dependence Matrix [24]

GLDM represents the dependencies of one gray level on other gray levels. It is defined as a set of connected voxels within distance *∂* dependent on a central voxel. A voxel with a gray level *i* is dependent on another voxel of gray level *j* if

(3) $$\begin{array}{c} \left|i-j\right|\leq \gamma . \end{array}$$

**Figure 11:**
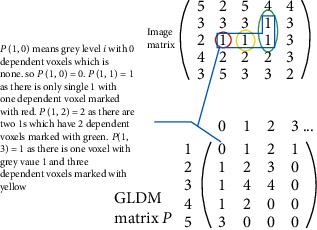
GLDM. Different color schemes were used to track GLDM procedure.

The (*i*, *j*)^th^ element of GLDM *P*(*i*, *j*) represents how often a voxel with the gray value *i* coexists with its dependent voxel having gray level *j* occurs in the whole brain image.  Figure 11 describes how GLDM is obtained from brain MRI with *n* = 5, i.e., 5 discrete gray levels, *γ* = 0, and *∂* = 1. The GLDM columns start from 0, and it can go to any finite number of dependent voxels.

The above GLDM is used to extract the following features:

- Small dependence significance
- Large dependence significance
- Gray-level heterogeneity
- Dependence heterogeneity
- Dependence heterogeneity normalized
- Gray-level deviation
- Dependence deviation
- Entropy of dependency
- The low gray-level significance
- The high gray-level significance
- Small dependency and low gray-level significance
- Small dependency and high gray-level significance
- Large dependency and low gray-level significance

#### 2.3.8. Decreasing Gray-Level Matrix (Novel Filter)

**Figure 12:**
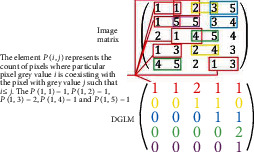
DGLM. Different color schemes were used to track DGLM procedure.

We propose a novel filter matrix to improve feature set. The *p*(*i*, *j*) | ∅, *δ* pixel of DGLM represents the occurrence of the pixel with intensity *i* and pixel with intensity *j* such that *i* ≤ *j*.  Figure 12 depicts and obtains a DGLM from the image with Ø = 0 and *δ*=1. Colors are used to track down the location of pixels for which the condition *i* < *j* holds true.

The DGLM is used to extract the following features in four directions, i.e., 0, 45, 90, and 135. Then, the average is taken to get the summary of the following features:

- Energy
- Mean
- Absolute mean deviation
- Skewness
- Kurtosis
- Entropy
- Autocorrelation

### 2.4. Feature Selection

Feature selection is the process of evaluating and selecting the most important features from the set of all features depending on their contribution to the machine learning task at hand. This process helped us to select the features with the highest predictive relevance to our classification task. This in turn also helps to eliminate redundant features.

In our study, we focused on tree-based classification methods. These methods have intrinsic feature selection methods. Using these intrinsic methods, we found the feature relevance for that each classifier.

**Figure 13:**
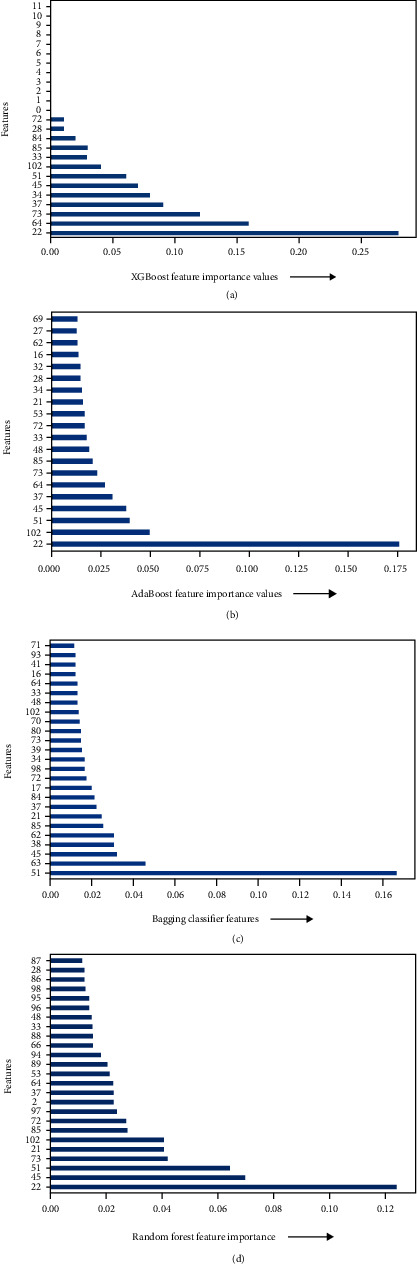
(a) Feature importance prediction by XGBoost. (b) Feature importance prediction by AdaBoost. (c) Feature importance prediction by bagging. (d) Feature importance prediction by random forest.

Figure 13(a) denotes that the novel feature “first order mean of DLGM” has the highest predictive power for XGBoost classifier; hence, it is the most important feature for this classifier. The other important features for XGBoost classifier are as follows:

- Decreasing Gray-Level Matrix feature first-order mean 0.26
- Gray-Level Dependence Matrix feature high gray-level emphasis 0.16
- Gray-Level Run Length Matrix feature gray-level run emphasis 0.14
- Gray-Level Cooccurrence Matrix feature correlation 0.08
- Gray-Level Cooccurrence Matrix feature cluster shade 0.05
- Decreasing Gray-Level Matrix feature information measure of correlation

Figure 13(b), denotes that the novel feature “first order mean of DLGM” has the highest predictive power for AdaBoost classifier; hence, it is the most important feature for this classifier. The other important top five features for AdaBoost classifier are as follows:

- Decreasing Gray-Level Matrix feature first-order mean 0.17
- Neighbouring Gray Tone Difference Matrix feature busyness 0.05
- Decreasing Gray-Level Matrix feature maximal correlation coefficient 0.04
- Decreasing Gray-Level Matrix feature information measure of correlation 0.035
- Gray-Level Cooccurrence Matrix feature correlation 0.03

Figure 13(c) denotes that the novel feature “Maximal Correlation coefficient of DLGM” has the highest predictive power for bagging classifier; hence, it is the most important feature for this classifier. The other important top five features for bagging classifier are as follows:

- Decreasing Gray-Level Matrix feature maximal correlation coefficient 0.16
- Gray-Level Dependence Matrix feature large dependence emphasis 0.05
- Decreasing Gray-Level Matrix feature information measure of correlation 0.03
- Gray-Level Cooccurrence Matrix feature difference average 0.03
- Gray-Level Dependence Matrix feature high gray-level emphasis 0.03

Figure 13(d) denotes that the novel feature “first order mean of DLGM” has the highest predictive power for random forest classifier; hence, it is the most important feature for this classifier. The other important features for random forest classifier are as follows:

- Decreasing Gray-Level Matrix feature first-order mean 0.12
- Decreasing Gray-Level Matrix feature information measure of correlation 0.07
- Decreasing Gray-Level Matrix feature maximal correlation coefficient 0.06
- Gray-Level Run Length Matrix feature high gray-Level run emphasis 0.05
- First-order mean absolute deviation 0.05

### 2.5. nsemble Learning Classifiers

“Ensemble learning is a machine learning paradigm where multiple learners are trained to solve the same problem. In contrast to ordinary machine learning approaches which try to learn one hypothesis from training data, ensemble methods try to construct a set of hypotheses and combine them to use” [29]. A good number of studies [30, 31] proved that the generalization capability of a set of learners is much greater than a single learner. Ensemble classifiers have been applied in diversified fields, e.g., cyber security, intrusion detection system, face recognition system, and traffic control systems. The concept of ensemble classification proceeds in two stages:

- Classifier generation
- Aggregation of results of these classifiers

There are three approaches to classifier generation and aggregation.

- Bagging
- Boosting
- Stacking

#### 2.5.1. Bagging

In this method, different training datasets are generated by resampling the training dataset, i.e., replacing some of the samples randomly. Suppose we have the following dataset: (4,5,6,7,8,9,10) and we have 5 classification algorithms. A different dataset is created by randomly resampling our data and passed to each classifier for training:

*Dataset for classifier 0*: (4,5,5,7,8,10,10) by replacing 6 with 5 and 9 by 10.

*Dataset for classifier 1*: (4,5,7,7,9,9,10) by replacing 6 by 7 and 8 by 9.

*Dataset for classifier 2*: (5,5,7,7,9,9,6) by replacing 4 by 5 and 10 by 6.

The results of all these classifiers are aggregated when taking predictions and inference time.

#### 2.5.2. Boosting

Boosting attempts to create chains of different classification algorithms. The chain with the best performance on training data is then used for inference, coming back to our previous example where we had our training dataset as (4,5,6,7,8,9,10) and 5 classification algorithms. If we are creating chains of 3 classifiers, we can create 10 such chains. A single chain of 3 classifiers is created in the following manner:

- A batch of training dataset is passed through classification algorithm 1, i.e., classifier 0

*Dataset for classifier 0*: (4,5,6,7,8,9,10)

(b) Based on the performance of classifier 0 on this training batch, the whole batch is redistributed. The incorrectly predicted samples (by classifier 0) from the training batch are chosen more often to create the training batch for classifier 1. In this manner, classifier 1 will try to improve on the mistakes done by classifier 0. This is true for each classifier in the chain

*Dataset for classifier 1*: (4,5,7,7,9,9,10) by replacing 6 by 7 and 8 by 9 as 7 and 8 was incorrectly predicted.

(c) The same process will be repeated for classifier 2

*Dataset for classifier 2*: (10,9,7,7,9,9,10) by replacing 4 by 10 and 5 by 7 as both 4 and 5 was incorrectly predicted.

In essence, boosting will create and choose the chain which is able to collectively give better results than other chains.

In this study, we have explored two boosting ensemble classifiers XGBoost and AdaBoost. As is evident from our results, the prediction accuracy with these classifiers is much higher than bagging classifiers.

#### 2.5.3. Stacking

Stacking is usually a 2 step approach. The classifiers in step 1 are known as base learners while the classifiers in step 2 are called stacking model learners. Each step is an ensemble of few classification algorithms. Predictions from the base learners are used as dataset for stacking model learners. Note that the predictions from base-level classifiers still maintain relationships with initial dataset which the stacking level classifiers can understand. The predictions from the stacking model learners are used at inference time.

## 3. Results

Along with accuracy, the most important metrics to analyze a biomedical machine learning study are sensitivity and specificity.

Sensitivity is the measure of true positives, which means accurate identification of patient with the disease. The test should have more true positives and minimum false negatives. False negatives mean we may miss out the positive identification of disease. Our study is a kind of screening test hence should have more sensitivity.  Table 1 shows highest sensitivity is 99.82% hence in accordance to screening test.

Specificity is the measure true negatives, which is the ability of a test to rule out the disease accurately. Target of study is to have minimum false positives. As the study is screening test, we can have false alarms and less specific. The specificity of our study is 97.01%.

The three metrics are measured with following formulae:

(i) Specificity = true negative outcomes/true negative outcomes + false positive outcomes 

(ii)  Sensitivity = true positive outcomes/true positive outcomes + false negative outcomes

(iii) Accuracy = true negative outcomes + true positive outcomes/true negative outcomes + false positive outcomes + true positive outcomes

### 3.1. Analyzing Different Ensemble Methods and Results

**Figure 14:**
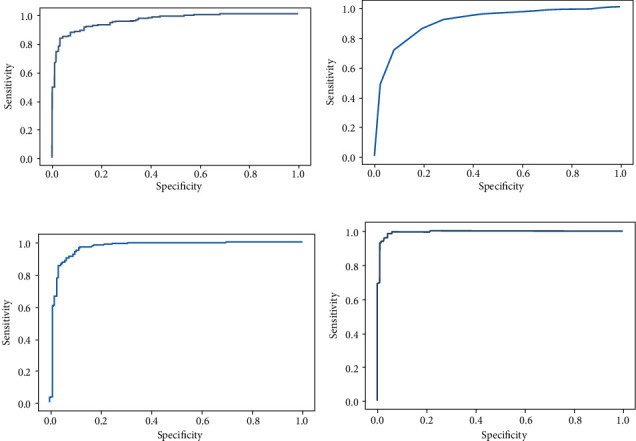
Area under the accuracy curve for different ensemble classifiers.

In our study, we observed that boosting ensemble learning classifiers such as AdaBoost and XGBoost perform better than bagging and randomized classifiers. Bagging classifiers and random forest classifiers yield almost the same accuracy of 87%. The results are listed in Table 1. The accuracy calculated from the area under the curve is depicted in Figure 14 for all four ensemble classifiers.

## 4. Conclusion

In this study, we have proposed to build a decision support system for radiologists in order to make fast and accurate decisions for early detection of brain degeneration by mapping CDR values to MRI images. The most important performance metrics in the field of computer-aided biomedical studies are sensitivity, specificity, and accuracy. Through this study, we have shown that better data collection and preprocessing (data augmentation and feature selection) along with gradient-boosted ensemble learning classifiers contribute to improvements in all 3 metrics.

Data is one of the most important factors for driving the accuracy of any study. In our study, we worked on the OASIS-3 dataset, which is a longitudinal dataset with 4096 MRI scans while earlier studies are performed on cross-sectional datasets with less than 500 MRI scans. This dataset also gives specific details about how the CDR value changes for a subject with respect to changes in the subject's MRI scan. Any machine learning system requires large amount of data to be optimally trained. In our study, we have also employed data augmentation techniques. Data augmentation resulted in our classifier being much more tolerant towards variance in the data; this prevents overfitting. Another major impact of data augmentation was the increase in dataset size from 4096 to 10000 MRI scans; this prevents underfitting. Mitigating overfitting and underfitting helps to achieve optimal accuracy on any dataset, irrespective of the classifier being used.

Our domain experts (Dr. Kunal Jain and Dr. Tanu) suggested that brain degeneration is not localized and affects the brain as a whole. As such, we have utilized whole brain volumes for our study and classification.

We experimented with Radiomics features and found that, for our data, the most promising features of

- GLCM are correlation, cluster shade, joint average, and cluster prominence
- GLRLM are gray-level run emphasis, short run high gray-Level emphasis, short run low gray-level emphasis, and gray-level variance
- NGTD Matrix is busyness
- GLDM are high gray-level emphasis and small dependence low gray-level emphasis
- GLSZM is small area low gray-level emphasis

Our study also proposes a novel texture filter DGLM. The features mean, information measure of correlation, maximal correlation coefficient, first-order entropy, and first-order skewness from novel DGLM improved the accuracy from 95.6% to 97.38%.

This study also reaffirmed the fact that ensemble learning classifiers are usually much more accurate than a single classification algorithm. The study observed that gradient-boosted classifiers do not suffer from overfitting and also help to reduce generalization error, hence improving accuracy, sensitivity, and specificity.

The study results have been compared to other different studies in this area as depicted in Table 2.

## Figures and Tables

**Table 1:** Sensitivity, specificity, and accuracy comparison of different ensemble classifiers.

Classifiers	Sensitivity	Specificity	Accuracy
XGBoost	99.82%	97.01%	97.38%
AdaBoost	94.91%	97.76%	97.21%
Bagging classifier	74.22%	90.07%	87.56%
Random forest	94.44%	87.07%	87.72%

**Table 2:** Comparison with similar studies.

Research study	Year	Dataset	Brain area	Classifier	Accuracy
Ahmad Chaddad [32]	2018	OASIS-1	Hippocampus Amygdala	Random forest Random forest	84.09%
				CNN	92.5%
Feng Feng [33]	2018	Local hospital data	Hippocampus	SVM	86.75%
Yupeng Li and Jiehui Jiang [34]	2019	Local hospital data	Hippocampus	SVM	91.5%
Kun Zhao [35]	Jan 2020	ADNI	Hippocampus	SVM	88.21%
Tao-Ran Li [36]	Dec 2020	ADNI	Right posterior and left superior cingulate Gyrus	SVM	95.9%
Current study proposed by us	2021	OASIS-3	Whole brain	Ensemble classifiers	
				XGBoost	97.38%
				AdaBoost	97.21%
				Bagging	87.56%
				Random forest	87.72%

## Data Availability

In this study, we used open source data. Data is available at https://www.oasis-brains.org/, the data was requested to Oasis-3 Brain team, and it provided the login and password to download the data; the same can be shared as and when needed.
